# Are Metabolites From the Gut Microbiota Capable of Regulating Epigenetic Mechanisms in the Human Parasite *Entamoeba histolytica*?

**DOI:** 10.3389/fcell.2022.841586

**Published:** 2022-03-01

**Authors:** Lotem Sarid, Serge Ankri

**Affiliations:** Department of Molecular Microbiology, Ruth and Bruce Rappaport Faculty of Medicine, Technion, Haifa, Israel

**Keywords:** *Entamoeba histolitytica*, gut mcirobiota, metabolites, epitranscriptome/epigenome, parasite

## Abstract

The unicellular parasite *Entamoeba histolytica* inhabits the human gut. It has to adapt to a complex environment that consists of the host microbiota, nutritional stress, oxidative stress, and nitrosative stress. Adaptation to this complex environment is vital for the survival of this parasite. Studies have shown that the host microbiota shapes virulence and stress adaptation in *E. histolytica*. Increasing evidence suggests that metabolites from the microbiota mediate communication between the parasite and microbiota. In this review, we discuss the bacterial metabolites that regulate epigenetic processes in *E. histolytica* and the implications that this knowledge may have for the development of new anti-amebic strategies.

## Introduction

*Entamoeba histolytica* is a parasitic unicellular organism that causes amebiasis, a disease of the intestinal tract. African and Central American countries with poor sanitation have higher rates of infection. In 2010 it was evaluated that *E. histolytica* causes 55,500 deaths and 2.237 million disability-adjusted life years (Turkeltaub et al., 2015). Food or water that contains *E. histolytica*’s cysts (the infective form) is the source of the infection. Cysts undergo excystation in the small intestine and trophozoites (the invasive form) are released. These trophozoites will then migrate to the large intestine, where they can colonize, or undergo encystation and be released in the feces. A majority of infections are asymptomatic, but for unknown reasons, the trophozoites can become highly virulent and can cause invasive disease (10%) (Chou and Austin, 2021). The development of vaccines targeting important *E. histolytica* antigens, such as Gal-lectin, the serine-rich protein and the 29 kDa-reductase antigen, has resulted in partial protection against *E. histolytica* infection in animal models (Quach et al., 2014). There is currently no vaccine for amebiasis. Among the treatment options for amebiasis, metronidazole is the first line of defense, but some side effects, such as diarrhea and anorexia, have been reported (Quach et al., 2014) (Leitsch, 2019). In the lab, metronidazole-resistant strains of *E. histolytica* have been developed, suggesting that such strains could emerge in the field as well (Wassmann et al., 1999). A population of 10^14^ microorganisms inhabits the large intestine. The gut bacteria is associated with unique combinations that are influenced by the physiological conditions within the intestine; most of these are commensal (Lynch and Pedersen, 2016). Trophozoites feed on gut bacteria, and the bacteria are able to influence the virulence of *E. histolytica* (for a recent review see Ankri, 2021). As an example, cultivating *E. histolytica* with *E. coli* O55 can boost *E. histolytica*’s virulence which depends on contact between the amoeba and bacteria (Bracha and Mirelman, 1984). Moreover, *E. histolytica* trophozoites were more resistance to oxidative stress (OS) after being incubated with *E. coli* O55 (Varet et al., 2018). Infection with *E. histolytica* can cause dysbiosis characterized by a decrease in *Lactobacillus* and *Bacteroides* and an increase in Bifidobacterium (Verma et al., 2012). *E. histolytica* must be capable of adapting to the changing environment in order to survive. Epigenetics deals with the study of changes in gene expression without altering DNA sequence. Epigenetic variation is able to respond to environmental shocks faster than genetic variation derived from evolutionary change (Prentis et al., 2008). A growing body of evidence supports the role of the gut microbiota in the development of pathological conditions, including cancer, through metabolites produced by the microbiota (Zhang et al., 2021). These metabolites have also a direct effect on the innate immune response of the host against *E. histolytica*. For example, deoxycholic acid produced by *Clostridium scindens* protects mice from *E. histolytica* infection by affecting granulocyte-monocyte progenitors levels in the bone marrow through epigenetic regulation of the level of intestinal neutrophils (Burgess et al., 2020). In contrast, the impact of microbiota metabolites on parasites is poorly understood. Our review discusses some gut microbiota metabolites and their role in the regulation of epigenetic events in *E. histolytica*.

## Current Knowledge of the Epigenetic Mechanisms Characterized in *Entamoeba* Parasites

### Histone Modifications

An earlier review of the chromatin organization, histone modifications, and their roles in *Entamoeba* virulence and differentiation has been reported (Tovy and Ankri, 2010). As a result of difficulties in getting pure histones from *Entamoeba* parasites, development on post-translational modifications in these histones is relatively modest (Lozano-Amado et al., 2016).

#### Histone Methylation

The methylation of histone tails occurs on arginine and lysine residues. Lysine methylation in *E. histolytica* histones regulates epigenetic regulation in this parasite. Inactivation of genes expression was linked to the demethylation of lysine 4 of histone H3 and the dimethylation of lysine 27 of histone H3 (Anbar et al., 2005) (Foda and Singh, 2015). Four expressed and catalytically active *E. histolytica* histone lysine methyltransferases (EhHKMTases) have been characterized (Borbolla-Vazquez et al., 2016). Two of these EhHKMTases, EhHKMT2 and EhHKMT4, may in addition to catalyze epigenetic marks, methylate proteins involved in phagocytosis. EhPRMT1 is an enzyme that catalyzes dimethylation of arginine 3 of histone H4 in *E. histolytica* (Borbolla-Vazquez et al., 2015). The importance of this epigenetic mark on the biology of *E. histolytica* is still not understood. The activity of histone demethylase in *E.histolytica* has not been adequately studied to date. Arginine residues can be demethylated by peptidyl arginine deiminase 4, which reverses methylation by converting arginine into citrulline (Cuthbert et al., 2004). A BLAST search of the genome of *E.histolytica*, however, found no homolog for this enzyme. However, an analysis of the *E. histolytica* genome reveals that there are three genes that encode SWIRM-domain proteins. SWIRM domains are found in lysine-specific demethylase 1, a protein that helps remove methyl groups from lysine 4 of histone 3 H3K4 (Forneris et al., 2006). Another large group of histone demethylases are the Jumonji C (JmJC) domain-containing enzymes (Cloos et al., 2008). To remove methyl groups, these enzymes undergo hydroxylation through the use of Fe2+ and oxygen. Interestingly, a search of the AmoebaDB database for Jumonji domain containing proteins revealed 28 candidates in Acanthamoeba and Naegleria parasites, but none in *Entamoeba* parasites. The oxygen-dependent mechanism of methyl group removal may not be compatible with the physiology of this anaerobic parasite.

#### Histone Acetylation

Detection of histone acetylation in *Entamoeba* parasites has largely been conducted by immunodetection (Ramakrishnan et al., 2004; Isakov et al., 2008), including more recent evidence of acetylation of lysines 5, 8, 12 and 16 in histone H4 of *E.histolytica* (Lozano-Amado et al., 2016). Histone acetylation and deacetylation are regulated by enzymes called histone acetylases (HATs) and histone deacetylases (HDACs). HATS are classified by their subcellular location within the nucleus and cytoplasm (Lee and Workman, 2007), as well as by their sequence and structure into several distinct families. GNAT (Krtenic et al., 2020) and MYST (Thomas and Voss, 2007) are the most recognized of these families. HDACs fall into four classes: HDACs (class I and II), NAD + -dependent deacetylases SIR2 (class III), and HDAC11 (class IV) (Park and Kim, 2020). The amoeba genome has so far been found to have a GNAT and MYST HAT, as well as a class I HDAC, although it is possible that other unidentified HATs and HDACs may be present as well (Ramakrishnan et al., 2004). *Entamoeba invadens*, a parasitic reptile used as a model system for studying development, has two class I HDACs that are homologous to human HDAC3. Among these HDAC3s, HDAC3-2 has the potential to play a role in the encystation by regulating gene expression involved in cyst wall formation (Lozano-Amado et al., 2020).

Histone acetylation is involved in *Entamoeba* parasite physiology as demonstrated by studies using trichostatin A (TSA), a powerful inhibitor of HDAC class I and II (Yoshida et al., 1990). TSA causes hyperacetylation of histones H4, inhibits encystation of *E. invadens* and downregulated the expression of enzymes involved in the formation of the cyst wall which suggest that encystation is control by the level of histone H4 acetylation (Lozano-Amado et al., 2020). On the other hand, TSA induces the expression of cyst wall-synthesis proteins like chitin synthase or the encystation-specific glycoprotein Jacob (Ehrenkaufer et al., 2007). A different response to TSA between *E.histolytica* and *E.invadens* could be related to each organism’s adaptation to living in its host.

### DNA Methylation

Mammals and other vertebrates methylate DNA in the C5 position of cytosine (m5C) mainly within CpG dinucleotides. The enzymes that catalyzed the formation of this modification are methyl 5-cytosine DNA methyltransferase (Dnmts) and S-adenosylmethionine is their cofactor. DNA methylation pattern maintenance is carried out by Dnmt1, while new DNA methylation occurs by Dnmt3a and B (Lyko, 2018).

The Dnmt2 protein is a member of a large family of proteins found in almost all species. Their activity as tRNA methyltransferases was first described in 2006 (Goll et al., 2006), and has been well recognized ever since (Jeltsch et al., 2017). Although Dnmt2 enzymes methylate tRNA molecules, they use a catalytic mechanism that is similar to that of DNA methyltransferases (Jurkowski et al., 2008). There is a lot of controversy regarding their ability to methylate DNA. The contradictory reports regarding the fruit fly *Drosophila melanogaster* illustrate this controversy (Phalke et al., 2009; Schaefer and Lyko, 2010; Dunwell and Pfeifer, 2014; Deshmukh et al., 2018). Experimental evidence supports Dnmt2 as a DNA MT enzyme in some parasites including *E.histolytica*, *Schistosoma mansoni* and more recently *Plasmodium falciparum* (Fisher et al., 2004; Geyer et al., 2011; Hammam et al., 2021). Dnmt2-mediated DNA methylation is likely to be involved in the control of repetitive DNA element which are considered genomic parasites (Harony et al., 2006; Lavi et al., 2008; Geyer et al., 2011).

### tRNA Methylation

Epitranscriptomics is field that deals with RNA modification. More than 150 post-synthetic RNA modifications are known to date, addressing all RNA species, which are catalyzed by more than 50 different RNA modifying enzymes (Boccaletto et al., 2018; Frye et al., 2018). tRNA modifications were found to take part in cell biology processes such as in tRNA stability, cellular stress response, drugs resistance and cell growth (Emilsson et al., 1992; Alexandrov et al., 2006; Begley et al., 2007; Netzer et al., 2009; Thompson and Parker, 2009; Motorin and Helm, 2010; Schaefer et al., 2010; Schimmel, 2018; Masuda et al., 2019). *E. histolytica* genome encodes for many tRNA modifying enzymes including 13 methyl transferases that belong to the NSUN and Dnmt2 family (Table 1). In mammalian, NSUN family consists of seven members, designated as NSUN1-7. Among these seven NSUN members, NSUN1, NSUN4 and NSUN5 could catalyze m5C on rRNA (Metodiev et al., 2014; Bourgeois et al., 2015); NSUN2, NSUN3 and NSUN6 are tRNA:m5C MTases (Brzezicha et al., 2006; Haag et al., 2015; Van Haute et al., 2016). Meanwhile, NSUN2 could also catalyze m5C on mRNA, vault RNA, microRNA and mitochondrial tRNA (Hussain et al., 2013; Yang et al., 2017; Shinoda et al., 2019). NSUN7 could catalyze m5C on enhancer RNA (Aguilo et al., 2016). The biological functions of some mammalian NSUN members have been investigated, revealing roles in protein biosynthesis, cell proliferation and differentiation, and organ development (Chi and Delgado-Olguin, 2013). Concomitantly, aberrant expression of several NSUN members are closely related to diseases (Bohnsack et al., 2019; Chellamuthu and Gray, 2020). In *E. histolytica*, nothing is known about these modifications or any of these enzymes and their roles during stress response in the parasite except for 5-methylcytosine (m5C) at position 38 of tRNA^Asp^(GUC) which is catalyzed by Ehmeth, a Dnmt2-type MTase (Tovy et al., 2010). A recent review about Dnmt2 shows that the effect of this enzyme on tRNA methylation are multiple (Jeltsch et al., 2017) and it includes an effect on protein translation (Tuorto et al., 2012) and on the production of specific stress-induced tRNA-derived small RNAs. *E. histolytica* is capable of responding to changes in its surrounding glucose concentration: short term glucose starvation (12 h) led to the accumulation of enolase, a glycolytic enzyme, in the nucleus. Enolase interacted with the catalytic site of Ehmeth, subsequently inhibiting its tRNA MTase activity (Tovy et al., 2010). Overexpression of Ehmeth confers resistance to oxidative stress (OS) (Fisher et al., 2006) and nitrosative stress (NS) (Hertz et al., 2014). Ehmeth-mediated resistance to NS is associated with 1) high levels of tRNA^Asp^(GUC) methylation, 2) persistence of protein synthesis under conditions of NS, and 3) the specific expression of proteins which are involved in protein translation, protein transport, vacuolar sorting signaling, and resistance to OS and NS, such as alcohol dehydrogenase 2 (ADH2) and peroxyredoxin (Hertz et al., 2014).

**TABLE 1:** A bioinformatic search reveals a variety of tRNA modification proteins in *E. histolytica* (Partial list).

Protein name	Accession number
tRNA dihydrouridine synthase 1	XP_655221
tRNA dihydrouridine synthase 1	XP_655192
tRNA dihydrouridine synthase 3-like	XP_651736
RNA: NAD 2′ phosphotransferase (tRNA splicing)	XP_650634
RNA: NAD 2′ phosphotransferase (tRNA splicing)	XP_648371
tRNA nucleotidyltransferase	XP_654783
tRNA nucleotidyltransferase	XP_652076
tRNA delta(2)-isopentenylpyrophosphate transferase	XP_655777
tRNA adenosine deaminase subunit	XP_649973
DNA/tRNA C-5 methyl transferase (Ehmeth)	XP_655267
tRNA (uracil-5) methyl transferase	XP_650594
tRNA (uracil-5) methyl transferase	XP_650606
tRNA (1-methyladenosine) methyltransferase (poss)	XP_653764
tRNA (1-methyladenosine) methyltransferase (poss)	XP_650623
**tRNA cytosine methyltransferase (EHI_103830)Trm4/*Eh*NSUN2**	**XP_653353**
**tRNA cytosine methyltransferase (EHI_098500)**	**XP_656026**
**tRNA/rRNA C-5-methyltransferase (EHI_140970)**	**XP_654608**
N2N2-dimethylguanosine tRNA methyltransferase	XP_657464
tRNA methyltransferase subunit TRM5	XP_650095
tRNA methyltransferase subunit TRM8	XP_656291
tRNA methyltransferase subunit TRM8	XP_649121
tRNA methyltransferase subunit TRM10	XP 650920
queuine tRNA-ribosyltransferase	XP_656142
queuine tRNA-ribosyltransferase	XP_652881
D-Tyr-tRNA (Tyr) deacylase (poss)	XP_656041

Candidate tRNA:m5C MTases are highlighted in bold font.

### RNA-Mediated Silencing

In order to achieve RNA-mediated silencing in *E.histolytica*, several methods are currently used, including double-stranded RNA (Solis et al., 2009), short-hairpin RNA (Linford et al., 2009), *trans*-inactivation (G3 strain) (Bujanover et al., 2003), and trigger-induced RNAi (Morf et al., 2013).

DICER and RISC complex are used in the first two methods (Paturi and Deshmukh, 2021). Despite lacking a gene matching the canonical Dicer structure, *E. histolytica* does express an RnaseIII protein (EHI_068740) (Abed and Ankri, 2005; Pompey et al., 2015). Of the three AGO genes, only one (EHI_125650) is highly expressed in the parasite (Zhang et al., 2011). Small RNAs of 27nt are associated with the AGO protein, providing evidence for a RNA-mediated silencing system in *E. histolytica* (Zhang et al., 2011).

G3 strain based gene silencing uses an *E.histolytica* strain that has been transfected with an upstream region of Ehap-a in order to silence expression of the amoebapore A gene (Bujanover et al., 2003). Even after removing the selectable marker, the silencing is stable, and the plasmidless gene-silenced clone G3 can be used to silence another gene. The presence of demethylated K4 in histone H3, a marker for inactive euchromatic regions, near the Ehap-a gene is consistent with transcriptional inactivation of that gene (Mirelman et al., 2006)

Small RNAs are used in trigger-induced RNAi to silence specific genes by sequence-specific silencing. Since the levels of antisense small RNAs correlate inversely with the mRNA expression levels of their cognate target genes, these small RNAs are thought to play an important role in gene silencing (Matthiesen et al., 2019). Demethylation of K27 in histone H3, a repressive histone mark, is enriched at genes silenced by RNAi (Foda and Singh, 2015).

### Possible Roles of Microbiota Metabolites in Shaping the Epigenetic Mechanisms in *E.histolytica*

#### Folate and S-Adenosylmethionine

Folate (or vitamin B9) is a central agent in the formation of SAM (Bailey and Gregory, 1999; Abbasi et al., 2018) by providing the methyl groups following the re-methylation of homocysteine to methionine (Kim, 2005; Krautkramer et al., 2017). SAM is a crucial cofactor participating in enzyme catalysis of many methyltransferases (MTases). In the host, SAM originates from methionine, an essential amino acid obtained through nutrition (Poirier et al., 2001). Folate is in part produced by the gut microbiota (Engevik et al., 2019). It has been suggested that dysbiosis of the gut microbiota can affect SAM levels and, therefore, modify MTases activity. Lactic acid bacteria naturally present in the human gut or probiotics are recognized for their ability to produce folate (Pompei et al., 2007). These bacteria may have an impact in the overall level of folate in the host and consequently on the level of SAM. *E. histolytica* requires folate for growth in axenic conditions (Diamond et al., 1978)*,* possibly indicating the parasite is dependent upon the gut microbiota for folate production. L-cysteine pool in the gut depends on dietary habits (Bauchart-Thevret et al., 2009), uptake by the host (Banjac et al., 2008), *de novo* synthesis or degradation by the gut microbiota into hydrogen sulfide (Neis et al., 2015; Braccia et al., 2021). Deprivation L-cysteine in *E. histolytica* results in a significant decrease in SAM levels (Husain et al., 2010) which may affect the activity of MTases. These data strongly suggest that the gut microbiota influences the level of SAM in *E. histolytica* and, as a result, the activity of its MTases.

It is still not completely understood how tRNA^Asp^(GUC) methylation regulates the synthesis of these proteins. However, this regulation might depend on the SAM level dictated by the gut microbiota. An example to support this notion has been provided by changes in DNA methylation observed in cocultivated human fetal and adult intestinal epithelial cells exposed to folate producing bacteria *Lactobacillus acidophilus* and *Bifidobacterium infantis* (Cortese et al., 2016; Qin and Wade, 2018). Recently, a new mechanism that links tRNA methylation to the gut microbiota activity has been revealed. This mechanism relies on queuine, a micronutriement produced by the gut microbiota.

#### Queuine

Queuosine (Q) and its glycosylated derivatives occur in position 34 of the anticodon of tRNA^Asp^, tRNA^His^ tRNA^Asn^ and tRNA^Tyr^ of eubacteria and eukaryotes except for *Saccharomyces cerevisiae* (Walden et al., 1982; Fergus et al., 2015). Q is highly conserved and found in plants, fishes, insects and mammals. While many bacteria can synthesize queuine (the nucleobase of Q) *de novo*, salvage of the prokaryotic Q precursors preQ_0_ and preQ_1_ has recently be reported (Yuan et al., 2019). Eukaryotes are not capable of Q synthesis and rely on salvage of the queuine base as a Q precursor either by nutrition or by the intestinal bacterial flora (Farkas, 1980; Katze et al., 1982; Ott et al., 1982). Queuine has been associated with neuroprotection (Richard et al., 2021) whereas absence of queuine is associated with better cancer cells growth and survival by promoting Warburg metabolism (Hayes et al., 2020). The tRNA-guanine transglycosylase (TGT) is the main enzyme responsible for the formation of Q in the anticodon loop position 34 of tRNA^Asp^, tRNA^His^, tRNA^Asn^ and tRNA^Tyr^. The enzyme exchanges G34 for the precursors. The cyclopentendiol moiety is synthesized at the level of tRNA from unknown precursors and enzymes in both eubacterial and eukaryotic species. The crystal structure of TGT from *Zymomonas mobilis* comprises an irregular (β/α)_8_ TIM barrel with a C-terminal zinc binding subdomain (Stengl et al., 2007)**.** In contrast to eubacterial TGT enzymes, all of which are homodimers, eukaryotic TGT enzymes, such as human TGT, are heterodimers and consist of a Q tRNA-ribosyltransferase 1 (QTRT1, eubacterial TGT homolog) and a Q tRNA-ribosyltransferase domain-containing 1 (QTRTD1) (Stengl et al., 2007; Chen et al., 2010). Both subunits are homologous to the bacterial domain QTRT; however, while QTRT1 should be catalytically active, the QTRTD1 subunit has lost residues important both for binding and catalysis. The source of transglycosylase activity of human TGT is the *h*QTRT1-*h*QTRTD1 heterodimer, while *h*QTRT1 and *h*QTRTD1 monomers do not exhibit any activity (Chen et al., 2010). Both *h*QTRT1 and *h*QTRTD1 co-localize in mitochondria (Boland et al., 2009). The crystal structure of *h*QTRTD1 (also called QTRT2) revealed that *h*QTRTD1 forms a homodimer with striking similarity to that of bacterial TGT (Behrens et al., 2018). Recently, the crystal structure of hTGT in its heterodimeric form and in complex with a 25-mer stem loop RNA has been established (Sievers et al., 2021). The detailed analysis of its dimer interface and interaction with a minimal substrate RNA indicates that one base only, guanine 34 or queuine, can simultaneously reside at the active site in support to a “ping-pong” mechanism that has already been proposed for *E.coli* TGT (Goodenough-Lashua and Garcia, 2003). Regarding hQTRTD1, the authors proposed that it could serve to anchor the TGT enzyme in the compartmentalized eukaryotic cell (Sievers et al., 2021). Based on the annotation of the *E. histolytica* genome, a homolog of *h*QTRT1 and *h*QTRTD1 exists in *E. histolytica*, namely *Eh*QTRT1 (XP_656142.1) and *Eh*QTRTD1 (XP_652881.1). *E.histolytica* tRNA-guanine transglycosylase (TGT) is a heterodimer composed of *Eh*QTRT1 and *Eh*QTRTD1 (Nagaraja et al., 2021). EhTGT is catalytically active and it incorporates queuine into *E. histolytica* tRNAs. The presence of Q in tRNA^Asp^_GUC_ stimulates its methylation by Ehmeth, a Dnmt2-type multisubstrate tRNA methyltransferase, at the C38 position. Queuine does not affect the growth of the parasite, it protects the parasite against oxidative stress (OS) and it antagonizes the negative effect that OS has on translation by inducing the expression of genes involved in OS response, such as heat shock protein 70 (Hsp 70), antioxidant enzymes, and enzymes involved in DNA repair. On the other hand, queuine impairs *E. histolytica* virulence determined in mouse model of amebic colitis by downregulating the expression of genes previously associated with virulence, including cysteine proteases, cytoskeletal proteins, and small GTPases. Silencing of EhTGT expression prevents the incorporation of queuine into tRNAs, impairs the methylation of C38 in tRNA^Asp^_GUC_, inhibits the growth of the parasite, impairs its resistance to OS and its cytopathic activity. Information about how Q is salvage by eukaryotic organisms is scanty. In mammalian cells, queuine is generated from Q- 5′-phosphate which suggests that salvage Q base is coming from tRNA degraded during the normal turnover process (Gunduz and Katze, 1984). In the green algae *Chlorella pyrenoidosa* and *Chlamydomonas reinhardtii* an enzymatic activity that catalyzes the cleavage of Q has been identified but its nature is not known (Kirtland et al., 1988). A search of genes that co-distribute with eukaryotic QTRT1 and QTRTD1 identified a potential Q salvage protein, DUF2419 (Zallot et al., 2014). The structural similarity of DUF2419 with DNA glycosylases suggests a ribonucleoside hydrolase activity. Indeed, genetic evidences support the role of DUF2419 as a Q salvaging enzyme in *Schizosaccharomyces pombe*, human, maize, and *Streptococcus thermophilus* (Zallot et al., 2014).

#### Short Chain Fatty Acids

Increasing evidences support the role of SCFA which are exclusively produced by the gut microbiota from the catabolism of carbohydrates of dietary origin in the regulation of global histone acetylation and methylation in the host (Krautkramer et al., 2016; Kelly et al., 2018). The abundances of SCFA depends on the composition of the microbiota with the dominant-SCFA producing bacteria being *Faecalibacterium prausnitzii* (Lopez-Siles et al., 2017) and *Roseburia intestinalis* (La Rosa et al., 2019). Acetate and propionate are important SCFA that inhibit histone deacetylases (HDACs) (Chen et al., 2003; Maslowski and Mackay, 2011) leading to histone decondensation, chromatin relaxation and active transcriptional state (Chen et al., 2003). Butyrate, another SCFA, inhibits HDACs by binding to Zn^+2^ that is located in their active site (Davie, 2003). Gut microbiota dysbiosis is associated with many intestinal and extra-intestinal disorders including bowel disease, metabolic syndrome, obesity, cardiovascular disease (Carding et al., 2015) and neurodegenerative disorders (Aho et al., 2021). Many of these disorders may have been triggered by a dysregulation of SCFA production consequent to dysbiosis (Tan et al., 2014). *E. histolytica* causes dysbiosis in the human gut by feeding on preferred bacteria like *Lactobacillus ruminus* (Iyer et al., 2019) and SCFA producing bacteria like *Bifidobacterium longum* (Iyer et al., 2019). Therefore, it is possible that *E. histolytica* infection change the level of SCFA in the host; however experimental evidences are needed to confirm this hypothesis.

SCFA have a direct effect on *E.histolytica* by promoting its encystation (Wesel et al., 2021). By consuming SCFA-producing flora, the parasite may control its entry into encystation through a modulation of its HDAC. It is important to note that SCFA have the opposite effect on the encystation of *Entamoeba invadens*, a reptile parasite (Byers et al., 2005). *Entamoeba* parasites may have evolved to respond differentially to SCFA produced by the microbiota of their respective host.

As mentioned earlier, repressive histone marks (demethylated K4 and K27 in histone H3) are present in the vicinity of silenced genes in *E.histolytica*. Consequently, one must speculate that SCFA affects gene silencing by inhibiting histone deacetylase activity. There is, however, a need to be cautious before jumping to conclusions, as reversal of the silencing seems to be resistant to TSA treatment (Bracha et al., 2003).

## Conclusion and Perspectives

**FIGURE 1:**
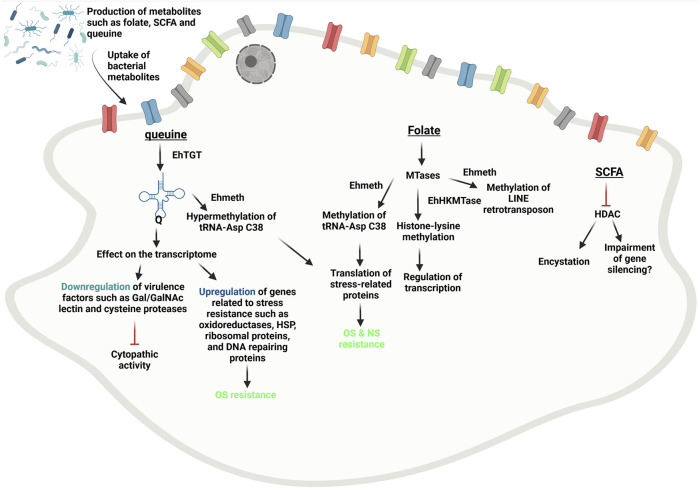
Legend summary of the effect of bacterial-metabolites on *E. histolytica* epigenome. Queuine incorporated into specific tRNA by EhTGT and lead to change in the transcriptome, OS resistance, hypermethylation of tRNAAspC38 and impaired virulence of *E. histolytica* trophozoites (Nagaraja et al., 2021). SCFA are HDAC inhibitors (Waldecker et al., 2008). SCFA promote encystation of *E. histolytica* (Wesel et al., 2021). Folate is critical for the formation of SAM which is used as cofactor of MTases (Abbasi et al., 2018). Lysine methylation in histones is catalyzed by EhHKMTases and demethylation of H3-lysine 4 is often indicative of inactivation of genes expression (Anbar et al., 2005). Ehmeth is a Dnmt2 homolog that can methylate LINE retrotransposon (Fisher et al., 2004; Harony et al., 2006) and tRNAAspC38 which is the favorite Ehmeth substrate (Tovy et al., 2010). Ehmeth activity is correlated to OS&NS resistance probably through the regulation of stress-related proteins translation (Fisher et al., 2006; Hertz et al., 2014). This document has be created with BioRender.com (accessed on December 21, 2021).

An increasing number of studies support an intricate relationship between the gut microbiota and *E. histolytica* (Burgess and Petri, 2016; Ankri, 2021) and significant changes in the gut microbiota has been associated with amebiasis (Rani et al., 2006; Verma et al., 2012). For example, the presence of *Prevotella copri*, in gut flora was associated with *E. histolytica* induced diarrheal disease in children (Ngobeni et al., 2017). These characteristics may serve as biomarkers for screening of amebiasis and prognosis. The gut microbiota impacts probably the manifestation and development of amebiasis through immunity (Burgess and Petri, 2016; Watanabe et al., 2017; Burgess et al., 2020), metabolism (Nakada-Tsukui and Nozaki, 2016), direct interaction with the parasite (Bracha and Mirelman, 1984; Padilla-Vaca et al., 1999; Galvan-Moroyoqui et al., 2008; Varet et al., 2018), metabolites that promotes stress resistance in the parasite (Shaulov et al., 2018; Nagaraja et al., 2021) and modulate epigenetic functions (this review). Fine-tuning the gut microbiota through diet or probiotics has been considered as a possible strategy to improve the efficacy and decrease the toxicity of treatment against cancer (for a recent review see (Zhou et al., 2021)). The same strategy has been considered for the prevention of amebiasis (Nagaraja and Ankri, 2019; Ankri, 2021). Some studies proposed to use probiotics to inhibit the binding of the parasite to intestinal mucosa surface (Rigothier et al., 1994; Mansour-Ghanaei et al., 2003) or to benefit from their amebicide activity (Varet et al., 2018). However, the idea of using probiotics and their metabolites to manipulate the epigenome of *E.histolytica* is just emerging. As summarized in Figure 1, we have discussed a number of metabolites that may be used for this purpose. By impairing the virulence of *E. histolytica*, queuine is a serious candidate. Yet, many questions remain about the choice of the probiotic queuine donor, the possibility to use queuine as postbiotic and the safety of this approach to human. Regarding safety, probiotics are generally considered harmless for immunocompetent individuals but sepsis in immunocompromised individuals or critically ill patients has been reported (Doron and Snydman, 2015). Finally, we need to be cautious in extrapolating the conclusions drawn about probiotics and their metabolites from animal experimentations to human.
